# Rapid Clinical Assessment to Facilitate the Triage of Adults with Falciparum Malaria, a Retrospective Analysis

**DOI:** 10.1371/journal.pone.0087020

**Published:** 2014-01-29

**Authors:** Josh Hanson, Sue J. Lee, Sanjib Mohanty, M. Abul Faiz, Nicholas M. Anstey, Ric N. Price, Prakaykaew Charunwatthana, Emran Bin Yunus, Saroj K. Mishra, Emiliana Tjitra, Ridwanur Rahman, Francois Nosten, Ye Htut, Richard J. Maude, Tran Thi Hong Chau, Nguyen Hoan Phu, Tran Tinh Hien, Nicholas J. White, Nicholas P. J. Day, Arjen M. Dondorp

**Affiliations:** 1 Mahidol Oxford Tropical Medicine Research Unit, Mahidol University, Bangkok, Thailand; 2 Global Health Division, Menzies School of Health Research, Darwin, Australia; 3 Centre for Tropical Medicine, Nuffield Department of Clinical Medicine, Churchill Hospital, Oxford, United Kingdom; 4 Department of Medicine, Ispat Hospital, Rourkela, Orissa, India; 5 Dev Care Foundation, Dhaka, Bangladesh; 6 Chittagong Medical College, Chittagong, Bangladesh; 7 National Institute of Health Research and Development, Ministry of Health, Jakarta, Indonesia; 8 Shaheed Sharwardhy Medical College, Dhaka, Bangladesh; 9 Shoklo Malaria Research Unit, Mae Sot, Thailand; 10 Department of Medical Research, Lower Myanmar, Ministry of Health, Yangon, Myanmar; 11 Oxford University Clinical Research Unit, Hospital for Tropical Diseases, Ho Chi Minh City, Vietnam; Centro de Pesquisa Rene Rachou/Fundação Oswaldo Cruz (Fiocruz-Minas), Brazil

## Abstract

**Background:**

Most adults dying from falciparum malaria will die within 48 hours of their hospitalisation. An essential component of early supportive care is the rapid identification of patients at greatest risk. In resource-poor settings, where most patients with falciparum malaria are managed, decisions regarding patient care must frequently be made using clinical evaluation alone.

**Methods:**

We retrospectively analysed 4 studies of 1801 adults with severe falciparum malaria to determine whether the presence of simple clinical findings might assist patient triage.

**Results:**

If present on admission, shock, oligo-anuria, hypo- or hyperglycaemia, an increased respiratory rate, a decreased Glasgow Coma Score and an absence of fever were independently predictive of death. The variables were used to construct a simple clinical algorithm. When applied to the 1801 patients, this algorithm’s positive predictive value for survival to 48 hours was 99.4 (95% confidence interval (CI) 97.8–99.9) and for survival to discharge 96.9% (95% CI 94.3–98.5). In the 712 patients receiving artesunate, the algorithm’s positive predictive value for survival to 48 hours was 100% (95% CI 97.3–100) and to discharge was 98.5% (95% CI 94.8–99.8).

**Conclusions:**

Simple clinical findings are closely linked to the pathophysiology of severe falciparum malaria in adults. A basic algorithm employing these indices can facilitate the triage of patients in settings where intensive care services are limited. Patients classified as low-risk by this algorithm can be safely managed initially on a general ward whilst awaiting senior clinical review and laboratory data.

## Introduction

Almost all patients with severe falciparum malaria will be managed in a resource-poor setting without prompt access to pathology and radiology services. While there are prognostic tools to guide the management of adults with malaria, most require laboratory data for calculation (table 1) [1], [2], [3].

**Table 1:** Prognostic tools used to predict outcome in adults with severe malaria.

	Score		
Variable	0 (normal)	1 (abnormal)	2 (very abnormal)
GCS	15	11–14	≤10
Base Deficit	<2	2 to <10	≥10
Plasma bicarbonate	≥24	15 to <24	<15
Respiratory rate	<20	20 to <40	≥40

**Malaria Score for Adults (MSA)** [1]:

1 [severe anaemia (haemoglobin <5 g/dL)]+2 [acute renal failure (serum creatinine >3 mg/dl)]+3[Respiratory distress (respiratory failure requiring ventilator)]+4 [cerebral malaria (unrousable coma)].

**Malaria Prognostic Index (MPI)** [2]:

4.5 (GCS <5)+1.5 (GCS 5–11)+1(Parasitaemia >315,000/µL)+2.5 (Plasma lactate >5 mmol/L)+1 (Serum bilirubin >58 µmol/L)+1.5 (Pigmented parasites >20%)−1.5 (Treatment with ACT).

**Coma Acidosis Malaria (CAM) score** [3]: MSA (0–10) is calculated using the 4 variables, classified as absent (0) or present (1) and multiplied by their coefficients.

MPI (0–10) is calculated using the 5 variables, classified as absent (0) or present (1) and multiplied by their coefficients.

CAM score (0–4) is calculated as the base deficit score (0–2) plus the Glasgow Coma Score (GCS; 0–2). Bicarbonate-based CAM score (BCAM) (0–4) is calculated as the bicarbonate score (0–2) plus the GCS (0–2). **Respiratory rate–based CAM score (RCAM)** (0–4) is calculated as the respiratory rate score (0–2) plus the GCS score (0–2).

The vital signs (temperature, blood pressure (BP), heart rate and respiratory rate) are simple to measure and routinely recorded in all hospitalisations. Other basic clinical indices, including level of consciousness, oxygen saturation, blood glucose level (BGL) and urine output have been proposed as additional “vital signs” [4]. Early recognition of a change in these clinical parameters can promptly identify the deteriorating patient and may improve outcomes [5], [6]. Given the necessary emphasis on the clinical assessment of adults with severe malaria in the resource-poor setting, we examined whether such clinical indices might play a role in patient triage and considered how they may reflect the underlying pathophysiology of the disease.

## Methods

Data was collected from all adults with strictly defined severe falciparum malaria enrolled in studies published by our research unit between 1996 and 2013. These studies were SEAQUAMAT, a multi-centre trial (n = 1050) comparing the efficacy of artesunate and quinine [7]; a Vietnamese randomised clinical trial (n = 560) comparing the efficacy of artemether and quinine [8]; a Bangladeshi series (n = 163) examining the prediction of a requirement for renal replacement therapy [9] (which consisted, in turn, of patients enrolled in studies examining the efficacy of N-acetylcysteine and levamisole as adjuvant therapy [10], [11]); and PRiSM, a Bangladeshi and Indian series (n = 28) that examined fluid resuscitation [12]. All studies received prospective ethical approval from the Oxford Tropical Research Ethics Committee and local ethical bodies. Prospective written informed consent was given by participants (or an attending caregiver) for their clinical records to be used in the original studies. Although consent was not specifically obtained for this retrospective review, all information had been de-identified prior to its analysis.

### Clinical Data

Study doctors collected the clinical data. Temperature was measured using tympanic thermometers with fever defined as a temperature ≥37.5°C and hypothermia as a temperature <36°C. Tachycardia was defined as a pulse rate ≥100 beats/minute and bradycardia as <60 beats/minute. BP was recorded using a manual sphygmomanometer; mean arterial pressure (MAP) was determined using the formula [(2×diastolic BP)+systolic BP]/3. Shock was defined as a systolic BP<80 mmHg with cool peripheries. Tachypnoea was defined as an observed respiratory rate ≥20 breaths/minute. Level of consciousness was determined using the Glasgow Coma Scale (GCS). BGL was measured via finger-prick using a glucometer, or from venous blood with a point-of-care device (iStat, Abbott Laboratories); hypoglycaemia was defined as a BGL <3.5 mmol/L and hyperglycaemia as a BGL ≥12 mmol/L. Oxygen saturation was recorded with pulse oximetry. Oligo-anuria on admission was defined as a urine output <10 ml in the first hour of hospitalisation after passage of a urinary catheter.

### Statistical Analysis

The relationship between death and the indices of temperature, BP, heart rate, respiratory rate, level of consciousness, shock (yes/no), BGL, oxygen saturation and oligo-anuria (yes/no) were assessed using a Student’s t-test, the Mann-Whitney U test, or the chi-squared test, as appropriate. To quantify the prognostic utility of the clinical indices, multivariate logistic regression was performed using a forward and backward stepwise approach, with fatal outcome as the dependent variable. Only variables with a value <0.05 were retained in the final model. Oxygen saturation was not included as a covariate in the multivariate model because data were only available from the two smaller studies (n = 185). Appropriate fit of the final model was confirmed using the Hosmer-Lemeshow goodness-of-fit test after grouping the data by predicted probabilities of death into 10 approximately equal-sized groups. Visual inspection of the univariate relationships showed that mortality increased as some of the variables approached both their upper and lower limits. Therefore, fractional polynomials were explored and included in the final model to allow for any significant non-linear relationships. Predictors that remained in the final model were also included in a random effects model to adjust for any potential differences by study. All analyses were carried out using statistical software (STATA, Version 10, StataCorp).

## Results

A total of 1801 patients were enrolled in the four studies, their baseline characteristics are presented in tables 2 and 3; 396 (22%) died, 217 (12%) within 48 hours of their hospitalisation. 712 (39.5%) patients were treated with artesunate, 805 (44.7%) with quinine and 284 (15.8%) with artemether.

**Table 2:** Baseline characteristics of the patients in the four studies.

		SEAQUAMAT	Vietnam	Bangladesh	PRiSM
**Number**		1050	560	163	28
**Age**	Years	28 (21–40)	30 (22–40)	35 (23–45)	36 (24–45)
**Male sex**	number (%)	787 (75)	425 (76)	130 (80)	19 (68)
**Anti-malarial therapy**		521 Artesunate 529 Quinine	284 Artemether276 Quinine	163 Artesunate	28 Artesunate
**Died before discharge**	number (%)	242 (23)	83 (14.8)	66 (40.5)	5 (17.8)
**Died in the** **1^st^ 48 hours**	number (%)	122 (50.4)	48 (57.8)	44 (66.7)	3 (60)
**Temperature**	°Celsius	37.8 (37–38.9)	38.5 (37.5–39.5)	38 (37.1–38.9)	37.8 (37.1–38.5)
**Shock**	number (%)	134 (12.8)	43 (7.7)	9 (5.6)	0
**MAP**	mmHg	79.2 (69.3–89.1)	76.7 (70–86.7)	79.9 (73.2–90.2)	87.5 (79–100.2)
**Heart Rate**	beats/minute	*	104 (96–120)	108 (96–120)	102 (93–114)
**Oxygen saturation**	%	*	*	96 (94–97)	98 (97–99)
**Respiratory Rate**	breaths/minute	25 (20–32)	28 (24–32)	30 (26–36)	30 (26–36)
**GCS**		11 (8–15)	10 (8–15)	8 (6–11)	13 (8–15)
**RCAM score**		2 (1–3)	3 (1–3)	3 (3–3)	2 (1–3)
**Glucose**	mmol/L	6.5 (5.4–8.3)	5 (3.6–7.3)	6.8 (5.3–10.1)	6.2 (5.7–9.9)
**Oligo-anuric**	number (%)	205 (19.5)	44 (7.9)	*	8 (28.6)
**Peripheral** **parasitaemia**	parasites/mL	66.3 (10–238)	90 (17.8–340)	138 (33.7–357)	14.9 (4–446)
**Blood Urea** **Nitrogen**	mmol/L	12.1 (7.1–24.3)	14.3 (7–27.1)	14.7 (9–26)	15 (11.4–24.5)
**Creatinine**	µmol/L	*	176 (128–300)	122 (79.6–202)	172 (105–273)
**Lactate**	mmol/L	*	3.4 (2.1–5)	4.7 (3.3–7.2)	3.1 (1.8–5)
**Base deficit**	mmol/L	4 (1–9)	5 (2–8.9)	8 (3–12)	9 (7–13)
**Haematocrit**	%	30 (22–36)	30 (24–36)	30 (24–36)	32 (25–38)

All numbers represent median (interquartile range) except where stated.

* Variable was not collected in this study.

MAP: Mean arterial pressure; mmHg: millimetres of mercury; GCS: Glasgow Coma Score; RCAM: Respiratory Coma Acidosis Malaria score; mmol/L: millimoles per litre; µmol/L: micromoles per litre.

**Table 3:** Simple clinical indices assessed in the studies and their association with survival to discharge.

		Number of observations	All	Survived n = 1405	Died n = 396	P
**Temperature**	°C	1794	38 (37.2–39)	38 (37.2–39)	37.8 (37–38.9)	0.01
**Shock**	number (%)	1800	186 (10.3)	127 (9)	59 (15)	0.001
**MAP**	mmHg	1786	77.4 (69.3–87.4)	76.7 (69.3–86.7)	79.2 (69.3–90)	0.02
**Heart Rate**	beats/minute	739 a	105 (96–120)	104 (95–120)	110 (104–124)	0.007
**Oxygen saturation**	%	185 b	96 (94–98)	96 (94–98)	95 (92–97)	0.01
**Respiratory Rate**	breaths/minute	1796	28 (22–32)	26 (22–32)	30 (24–36)	0.0001
**GCS**		1800	10 (8–15)	12 (8–15)	8 (6–11)	0.0001
**RCAM score**		1795	2 (1–3)	2 (1–3)	3 (2–3)	0.0001
**Abnormal glucose**	number (%)	1769	400 (22.6)	266 (19.3)	134 (34.6)	<0.0001
**Oligo-anuric**	number (%)	1638 c	257 (15.7)	155 (11.9)	102 (30.9)	<0.0001

All numbers represent median (interquartile range) except where stated.

a Heart rate was not recorded in SEAQUAMAT;

b Oxygen saturation was only measured in the two smaller studies;

c Oligo-anuria was not recorded in the Bangladeshi study. MAP: Mean arterial pressure; mmHg: millimetres of mercury; GCS: Glasgow Coma Score; RCAM: Respiratory Coma Acidosis Malaria score;

### Prognostic Value of Clinical Signs – Univariate Analysis

#### Temperature

**Figure 1:**
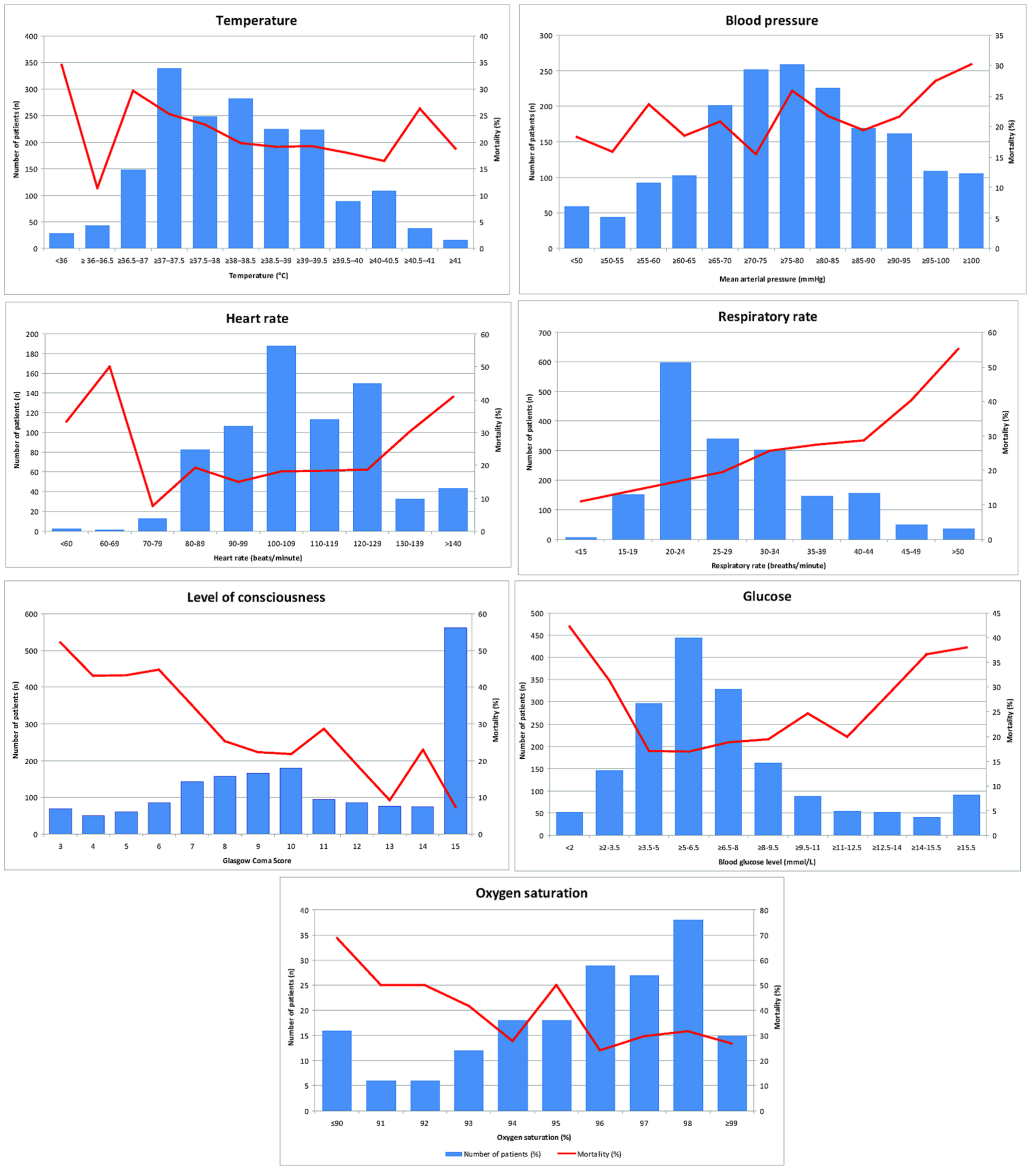
Relationship between simple clinical indices at presentation and in-hospital mortality.

**Figure 2:**
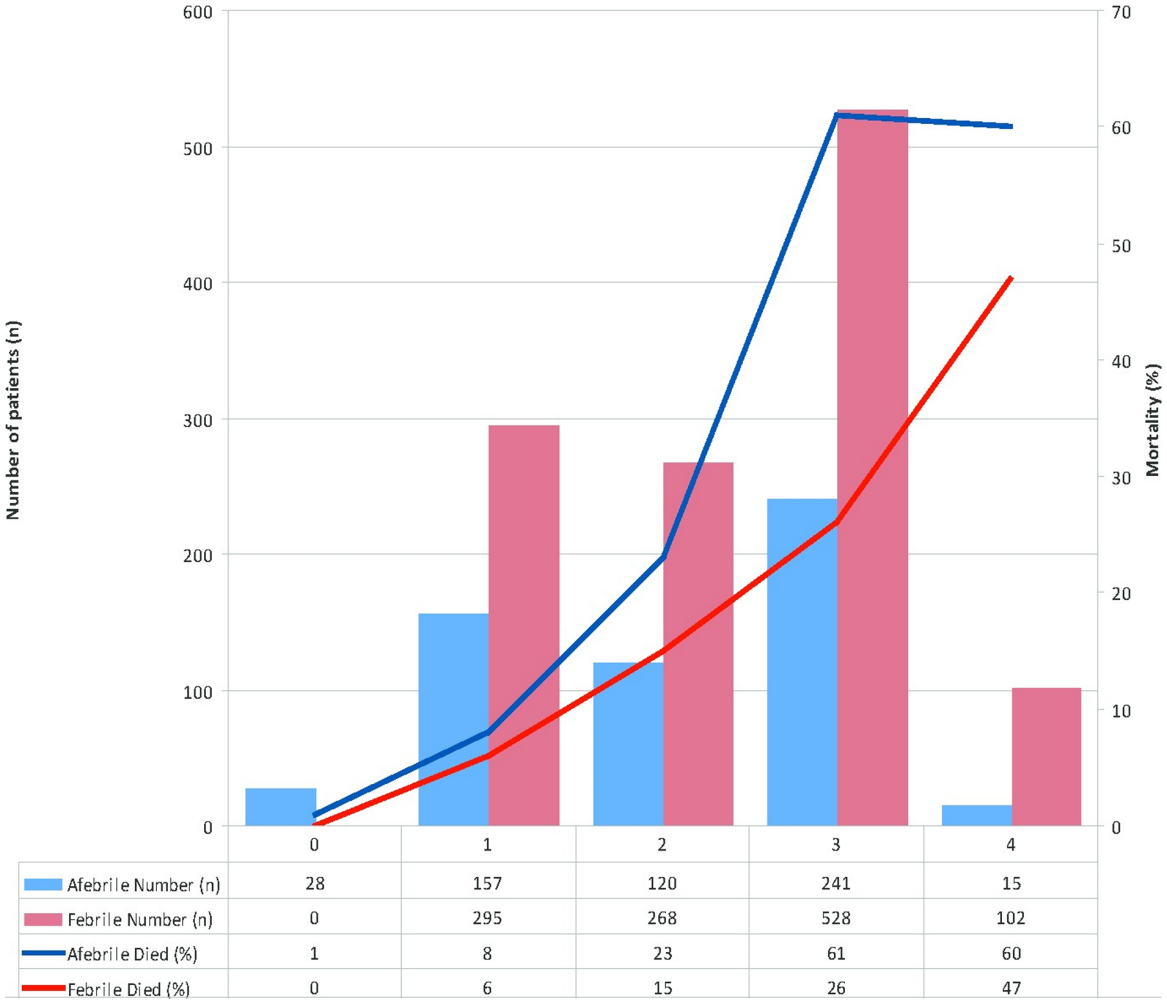
Relationship between fever and in-hospital mortality, stratified by disease severity (RCAM score). Febrile: tympanic temperature ≥37.5°Celsius, RCAM: as defined and calculated in table 1.

Fever was present on admission in 1233/1794 (69%) (figure 1). Overall 20% (247/1233) of patients febrile at enrolment died compared to 25.9% (145/561) of patients who were afebrile (odds ratio (OR) = 0.72, (95% confidence interval (CI)) 0.59–0.91; p = 0.006). This was despite the finding that febrile patients had more severe disease as determined using the RCAM score (p<0.001) (figure 2). Mortality was higher in hypothermic patients (34.5%), but it was an infrequent finding (29 (1.6%) of 1794 observations) and the association did not reach statistical significance (p = 0.11).

#### Heart rate

72% (532/739) of patients were tachycardic on enrolment and 3 (0.4%) bradycardic. Heart rate was associated with outcome (p = 0.007), however this finding was strongly influenced by the relatively few patients with a tachycardia ≥140 beats/minute (figure 1).

#### Blood pressure

Shock was not a common finding: 10.3% (186/1800) of patients were shocked on admission, with 59 (32%) dying compared with 21% (336/1614) of patients who were not shocked (p = 0.001). Even with shocked patients included in the analysis, mortality increased with rising MAP (p = 0.02) (figure 1). MAP was inversely correlated with GCS (r_s_ = −0.22, p<0.001).

#### Respiratory rate

Tachypnoea was present in 1417/1796 (78.9%); mortality rose steadily with respiratory rate (p = 0.0001) (figure 1).

#### Level of consciousness

1239/1800 (68.8%) had impaired consciousness (GCS<15) on admission. Mortality increased steadily as GCS declined (p = 0.0001) (figure 1).

#### Blood glucose

There was a non-linear relationship between BGL and death in univariate analysis (figure 1); this was the only variable for which a fractional polynomial term was added to the model (after which its relationship became statistically significant). 11.2% (199/1769) of patients were hypoglycaemic on admission, 68 (34%) of whom died. Patients hypoglycaemic on admission were more likely to die than those with a normal BGL (3.5–12 mmol/L) (OR (95% CI) 2.3 (1.7–3.2), p<0.001). Hyperglycaemia was as common as hypoglycaemia and was associated with a similarly poor outcome: 32.8% (66/201) of patients hyperglycaemic on admission died. Hyperglycaemic patients were also more likely to die than those with normal admission BGL (OR: 2.2, 95% CI 1.6–3, p<0.001).

#### Pulse oximetry

Pulse oximetry was only routinely available in the two smaller studies; however, in both oxygen saturation was inversely associated with mortality (p = 0.01) (figure 1).

#### Oligo-anuria

Oligo-anuria was associated with an increased requirement for renal replacement therapy (RRT) and greater mortality. Patients in SEAQUAMAT had limited access to RRT, which may have increased the associated mortality.

### Multivariate Analysis

To determine which variables had independent prognostic value, multivariate analysis was performed (table 4). Six independent predictors of death were identified: temperature (OR 0.8; 95% CI 0.71–0.9), respiratory rate (OR 1.04; 95% CI 1.03–1.06), GCS (OR 0.82; 95% CI 0.79–0.85), shock (OR 2.27; 95% CI 1.53–3.38) and oligo-anuria (OR 3.08; 95% CI 2.24–4.23); the sixth independent predictor, BGL, had a non-linear relationship.

**Table 4:** Significant predictors of in-hospital mortality on multivariate analysis.

	Odds ratio (95% CI)	Odds ratio stratified by study* (95% CI)
Temperature	0.83 (0.73–0.94)	0.85 (0.75–0.97)
Respiratory rate	1.04 (1.02–1.06)	1.04 (1.02–1.06)
Glasgow Coma Score	0.82 (0.79–0.85)	0.82 (0.79–0.85)
Oligo-anuria	3.09 (2.23–4.28)	2.90 (2.09–4.04)
Shock	2.27 (1.53–3.38)	2.21 (1.48–3.28)
Glucose	Non-linear relationship**	Non-linear relationship**

Adjusted odds ratios (95% CI), n = 1602.

* Odds ratios from random effects model, stratified by study;

** Mortality increases with both evolving hypo and hyperglycaemia.

### Use in Triage

**Figure 3:**
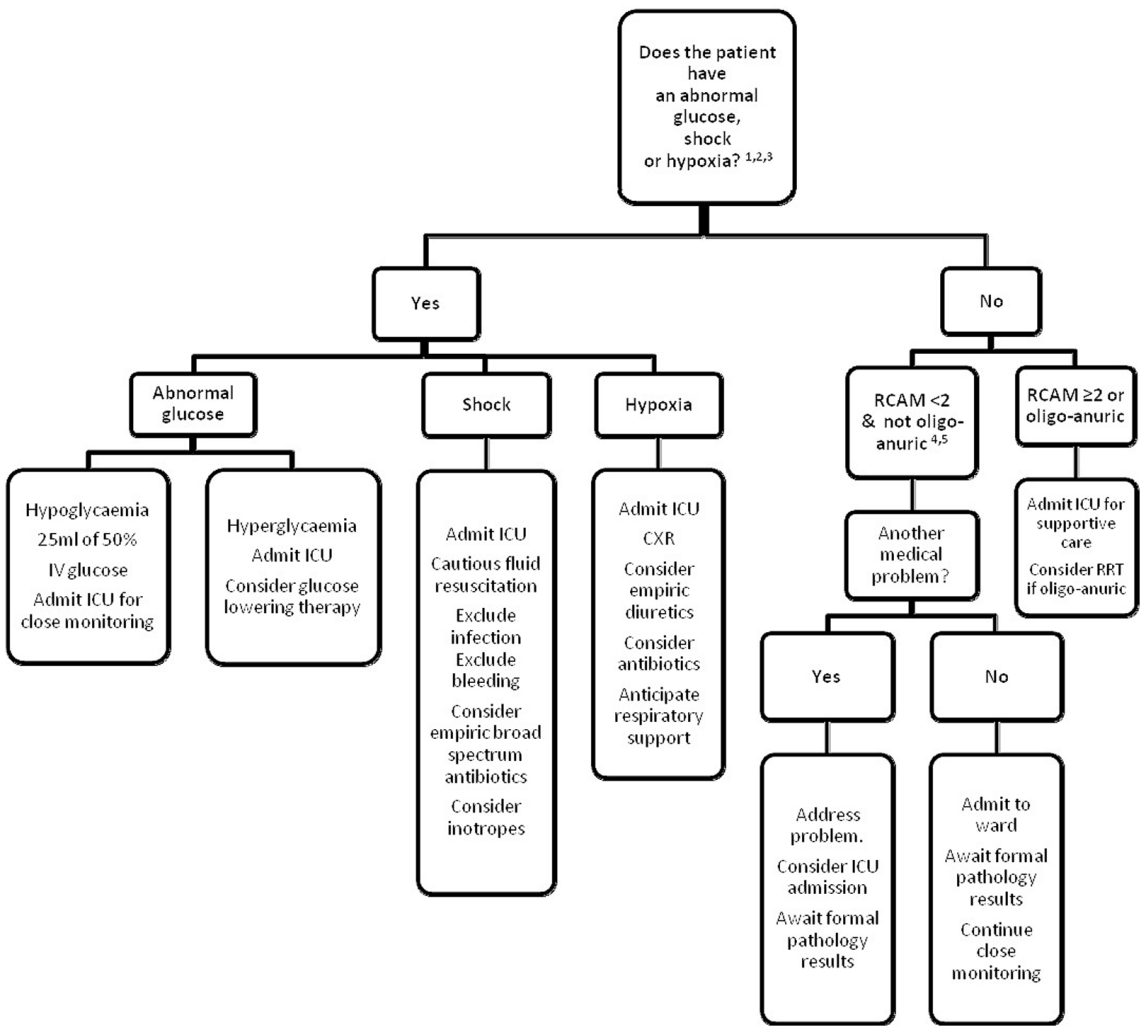
Proposed initial triage strategy for an adult with falciparum malaria in a resource-poor setting. ^1^Shock: systolic blood pressure <80 mmHg with cool peripheries. ^2^Hypoxic: SaO_2_≤95% using pulse oximetry. ^3^Abnormal blood glucose: blood glucose <3.5 mmol/L or ≥12 mmol/L using glucometer. ^4^RCAM (see table 1). ^5^Oligo-anuric: urine output <10 ml first hour after catheterization.

**Figure 4:**
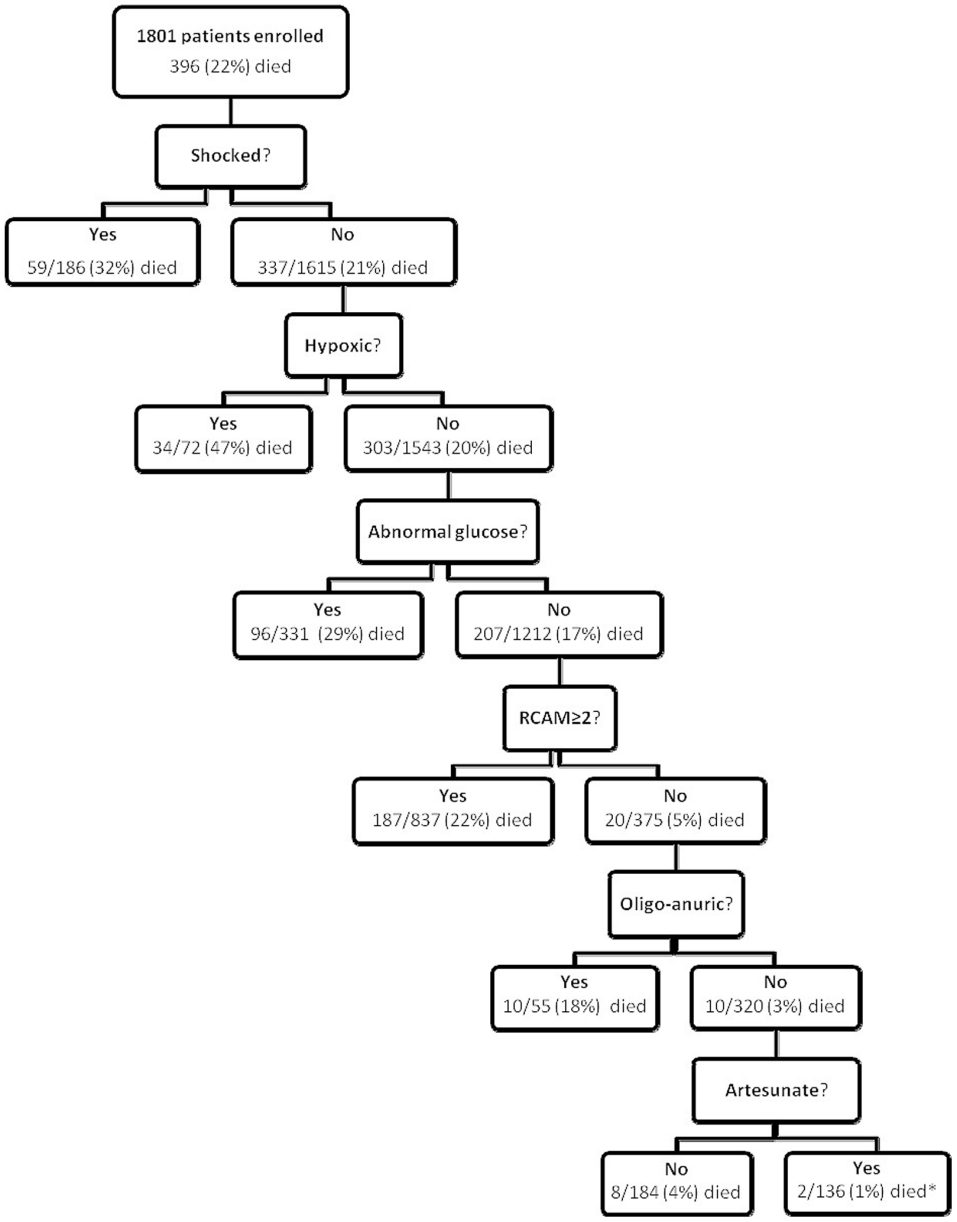
Performance of the clinical algorithm in the four studies. *Both died >48 hours after enrolment.

The data were used to construct a clinical algorithm to identify patients at greatest risk of dying (figure 3). Although there were insufficient data to use oximetry in the multivariate model, univariate analysis showed a strong association with mortality and therefore it was incorporated into the algorithm. Conversely, temperature was not employed as the absence of fever may also be a sign of health. The algorithm’s six simple clinical indices were shock, hypoxia (oxygen saturation ≤95%), abnormal BGL (<3.5 mmol/L or ≥12 mmol/L), respiratory rate and GCS (combined as a RCAM score with a cut-off at 2 (table 1)) and oligo-anuria. Cut-offs were determined by inspection of the data and extrapolation from common clinical practice. If data were missing, the patient was assumed not to satisfy that criterion. In applying the algorithm, the presence or absence of the signs was determined sequentially, reflecting typical bedside evaluation (figure 4).

Only 10/1801 (0.6%) patients without significant derangement of one of the six indices on admission died prior to discharge and only 2 (0.1%) died in the first 48 hours of their hospitalisation; 8 (80%) of the 10 deaths occurred in patients receiving inferior anti-malarial treatment (7 quinine, 1 artemether). Only 2/712 (0.3%) patients receiving artesunate, without significant derangement of one of the six indices on admission failed to survive to discharge; these patients died on the third and fourth day of their hospitalisation respectively. Thus simple clinical assessment on admission, in patients receiving standard anti-malarial therapy with artesunate, had a positive predictive value for survival at 48 hours of 100% (95% CI 97.3–100) and to discharge of 98.5% (95% CI 94.8–99.8) (table 5).

**Table 5:** Ability of the clinical algorithm to predict survival to discharge (patient assessed at admission).

	All patients			Only patients receiving artesunate		
Study	Number	Survival to discharge	Survival to 48 h	Number	Survival to discharge	Survival to 48 h
SEAQUAMAT	1050	96.8 (93.6–98.7)	99.6 (97.5–100)	521	98.4 (94.2–99.8)	100 (97.2–100)
Vietnam	560	96.6 (90.3–99.3)	98.8 (93.6–100)	0	n/a *	n/a *
Bangladesh	163	100 (71.5–100)	100 (71.5–100)	163	100 (71.5–100)	100 (71.5–100)
PRiSM	28	100 (29.2–100)	100 (29.2–100)	28	100 (29.2–100)	100 (29.2–100)
**Pooled**	**1801**	**96.9 (94.3–98.5)**	**99.4 (97.8–99.9)**	**712**	**98.5 (94.8–99.8)**	**100 (97.3–100)**

All numbers represent the positive prediction value and 95% confidence interval.

* No patient received artesunate in this trial.

**Figure 5:**
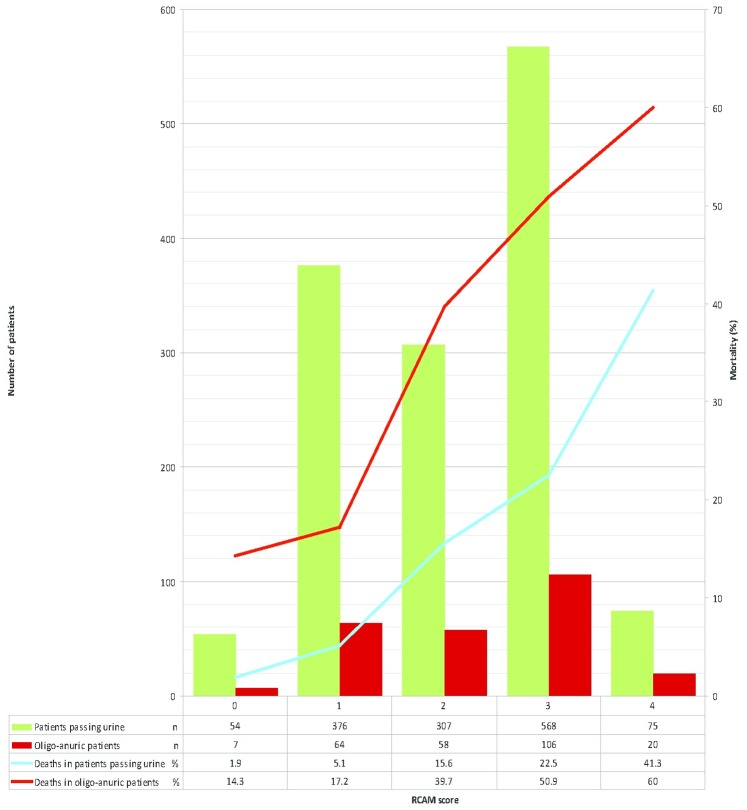
Relationship between oligo-anuria and in-hospital mortality, stratified by disease severity (RCAM score). RCAM: as defined and calculated in table 1. Oligo-anuric: urine output <10 ml first hour after catheterization.

A combination of an RCAM score <2 and the presence of oligo-anuria on admission identified 375/396 (94.7%) of deaths in the series (figure 5); 17 of the 21 unidentified deaths occurred in patients receiving inferior anti-malarial therapy and only 6 of the 21 died in the first 48 hours.

The provision of supportive care was most completely documented in SEAQUAMAT, although its availability was sometimes limited reflecting the trial’s poor resource-setting. Pulse oximetry was not routinely measured in SEAQUAMAT, but using the algorithm’s remaining five criteria 217/1050 (21%) were classified as low-risk; only 8 (3.7%) of these low-risk patients required dialysis, mechanical ventilation or inotropic support at any time during their hospitalisation compared with 150/833 (18%) of the high-risk patients (p<0.001).

## Discussion

While not obviating the need for repeated evaluation and confirmatory investigations, the presence of a few simple clinical indices on presentation can accurately identify those at risk of a complicated course, facilitating patient triage. Euglycaemic patients with a RCAM score <2, who are not shocked, hypoxic or oligo-anuric on admission and who are treated with parenteral artesunate are likely to have an excellent outcome and could safely be managed initially on a general ward while senior clinical review and laboratory data are awaited.

Coma, acidosis, and kidney injury have repeatedly been demonstrated to be the three strongest prognostic factors in adults with malaria [1], [2], [3]. Determination of their bedside correlates – GCS, respiratory rate, and oligo-anuria – at presentation identified almost 95% of the patients that would later die, a figure rising to over 99% if shock, hypoxia or deranged BGL were added to a clinical algorithm. Junior staff can perform this assessment at the bedside rapidly, efficiently identifying the patients most in need of care in a high-dependency setting.

Cerebral malaria is a striking syndrome and its implications for management have been appropriately emphasised, however the utility of a patient’s respiratory rate is generally underappreciated [13]. While respiratory distress is a World Health Organization (WHO) criterion for severe malaria, this focuses on the increased work of breathing seen predominantly in African children [14], rather than on the respiratory rate itself. Tachypnoea may indicate an underlying lactic acidosis or denote the important clinical syndromes of pulmonary oedema and aspiration pneumonia [15].

Acute kidney injury (AKI) occurs in up to half of adults with severe malaria, about a third of which is non-oliguric [9], [16]. Anuric AKI suggests established acute tubular necrosis and is frequently associated with late presentation and multi-system disease [12], [17]; less than 10% of anuric AKI responds to fluid and anti-malarial therapy. Here oligo-anuria was independently associated with a threefold increase in mortality. Promptly recognising cases of AKI that will not respond to simple measures expedites referral for RRT, which if delayed results in poorer outcomes [17].

The pattern of the clinical findings’ disturbance also provides insight into the pathophysiology of falciparum malaria, with potential implications for management. In the current series, patients who were febrile on admission were more likely to survive than afebrile patients, an observation that persisted in multivariate analysis. This was not the result of a harmful effect of hypothermia (which was infrequently seen) and echoes the findings in a series of 988 adults with severe malaria where the mean temperature on enrolment was also significantly higher in survivors [2]. This raises the possibility that fever may be a protective, adaptive response in severe malaria, as has been suggested by some, but not all, studies of severe bacterial sepsis [18], [19]. While an *in vitro* study showed that higher temperatures increased the cytoadherence of parasitised erythrocytes to vascular endothelium (the mechanism of sequestration) [20], other studies have shown that higher temperatures suppress parasite growth [21], [22]. African children with falciparum malaria receiving rectal paracetamol had a reduced cytokine response and a prolonged parasite clearance time, although the low dose used in this study (10–15 mg/kg, 6 hourly) may not have yielded therapeutic blood concentrations [23]. Since the classic fever pattern in malaria is periodic, the timing of a patient’s presentation influences their temperature on admission. Schizogony leads to fever and profound malaise and may precipitate the presentation of patients with milder disease [24]. However, this association is lost in severe disease and as these studies only enrolled patients meeting criteria for severe malaria this would not explain our findings; indeed the febrile patients actually had higher RCAM scores.

Young children have a high risk of febrile convulsions and require anti-pyretic treatment. In these studies anti-pyretic administration was incompletely documented precluding any conclusions; however the retrospective finding that febrile adults had a reduced case fatality rate warrants prospective investigation. Studies to define the role of paracetamol in severe malaria are ongoing (NCT01641289).

There are stark pathophysiological differences between malaria and bacterial infection. Shock is a relatively infrequent finding in patients with malaria compared to bacterial sepsis. Indeed shocked malaria patients are often demonstrated to have bacterial co-infection when sophisticated microbiological testing is available [25]. While hypotension helps define sepsis and is a powerful negative prognostic indicator [26], less than 20% of the patients in these four studies had an admission MAP<65 mmHg and mortality actually increased with rising MAP, similar to the findings in another series of Asian adults with falciparum malaria [2].

Why would higher – but by no means dangerously elevated – BP be associated with a poorer outcome? In PRiSM, MAP was >65 mmHg in 85% of patients despite the fact that all were concurrently hypovolaemic, some profoundly so [27]. The degree of microvascular sequestration – the pathological signature of falciparum malaria linked to outcome and organ failure - correlated with both the MAP and the systemic vascular resistance, most likely via an indirect mechanism of endothelial dysfunction and loss of vasodilatory tone [28]. The haemolysis seen in malaria results in the release of free haemoglobin leading to nitric oxide quenching which may exacerbate endothelial dysfunction [29].

These findings have implications for management: unlike bacterial sepsis, a “normal” BP in patients with severe malaria is not reassuring. Moreover, in contrast to severe sepsis where a key goal of resuscitation is fluid loading and inotropic support to maintain a MAP of >65 mmHg [26], the fact that most patients with severe malaria have a preserved BP supports a more conservative approach to rehydration, especially as liberal fluid loading is unequivocally harmful in this population, and has a limited effect on microvascular obstruction [12], [30].

The WHO uses hypoglycaemia to define severe malaria and its recognition and management is appropriately highlighted: a low BGL can be rapidly fatal, but has a simple remedy. In these studies many patients received treatment with quinine, an insulin secretagogue, before study enrolment. Hypoglycaemia was therefore common and was associated, as expected, with high mortality (although notably mortality remained elevated with hypoglycaemia up to 3.5 mmol/L, above the present WHO cut-off of 2.2 mmol/L [31]). However hyperglycaemia at presentation was equally common and was associated with a similar mortality. Some comatose patients may have received a glucose bolus prior to enrolment, as is recommended in settings where a BGL is not immediately available [31]. Yet controlling for GCS in multivariate analysis, mortality was still higher in hyperglycaemic patients, suggesting that, as in other critical care settings, this is a genuine association [32], [33].

In one study intensified insulin therapy reduced the mortality of critically ill hyperglycaemic surgical patients [34], although this strategy led to frequent severe hypoglycaemia in patients with bacterial sepsis [35] and increased mortality in a general ICU population [36]. While hyperglycaemia may exacerbate fluid and electrolyte depletion and have a negative effect on endothelial, immune and neurological function [37], [38], [39], some argue that by allowing vital tissues to utilise glucose independent of insulin some degree of permissive hyperglycaemia may be beneficial [40]. As artesunate replaces quinine as first-line anti-malarial therapy, the incidence of hypoglycaemia will decline, particularly in adults who experience the complication less frequently than children. With the emerging Asian diabetes epidemic, the recognition and management of hyperglycaemia - although more complicated - may become just as relevant. A prospective study may resolve the issue.

Our retrospective analysis has limitations. Although several clinical indices are emphasised, clearly laboratory and radiological investigations remain essential for optimal patient management. Similarly, the utility of selected clinical findings does not obviate the need for a detailed history and physical examination to identify other co-morbidities. Our analysis uses clinical findings present at admission which need to be followed sequentially; normal initial values do not preclude the possibility of subsequent complications. However it is noteworthy that while the ensuing medical care provided in the studies was not evaluated in detail, patients categorised as low-risk rarely required an escalation of care. The relative prominence of prognostic indicators in adults with severe malaria varies in different studies – this is related mainly to differences in the proportion of patients with severe disease, and definitions of severe disease, in different cohorts. In this series, only patients already enrolled in severe malaria studies were analysed, accordingly the proposed algorithm re-classified only 18% patients as low-risk; in a series of patients with milder disease an RCAM <2 alone had a PPV for survival to discharge of 98.4% [2]. Finally, these findings cannot be directly extrapolated to the paediatric population with malaria, although as the prognostic indicators are comparable, trends are likely to be similar [41].

## Conclusions

Clinical assessment of adults with falciparum malaria on admission to hospital, focusing on a few simple indices, is able to promptly identify those at risk of a complicated course, facilitating their triage to a high dependency setting. A clinical emphasis is more practical for health care workers who manage these patients in the resource-poor setting and can help guide management while more sophisticated assessment is awaited.

## Supporting Information

Checklist S1 PRISMA checklist.
(DOC)
